# Kernel recursive least square tracker and long-short term memory ensemble based battery health prognostic model

**DOI:** 10.1016/j.isci.2021.103286

**Published:** 2021-10-15

**Authors:** Muhammad Umair Ali, Karam Dad Kallu, Haris Masood, Kamran Ali Khan Niazi, Muhammad Junaid Alvi, Usman Ghafoor, Amad Zafar

**Affiliations:** 1 Department of Unmanned Vehicle Engineering, Sejong University, Seoul 05006, South Korea; 2 Department of Robotics and Intelligent Machine Engineering (RIME), School of Mechanical and Manufacturing Engineering (SMME), National University of Sciences and Technology (NUST) H-12, Islamabad 44000, Pakistan; 3 Department of Electrical Engineering, University of Wah, Wah Cantt 47040, Pakistan; 4 Department of Energy Technology, Aalborg University, Aalborg 9220, Denmark; 5 Department of Electrical Engineering, NFC Institute of Engineering and Fertilizer Research, Faisalabad 38090, Pakistan; 6 Department of Mechanical Engineering, Institute of Space Technology, Islamabad 44000, Pakistan; 7 Department of Electrical Engineering, University of Lahore, Islamabad Campus, Islamabad 44000, Pakistan; 8 Department of Electrical Engineering, The Ibadat International University, Islamabad 44000, Pakistan

**Keywords:** Computer systems organization, Energy engineering, Energy systems

## Abstract

A data-driven approach is developed to predict the future capacity of lithium-ion batteries (LIBs) in this work. The empirical mode decomposition (EMD), kernel recursive least square tracker (KRLST), and long short-term memory (LSTM) are used to derive the proposed approach. First, the LIB capacity data is split into local regeneration and monotonic global degradation using the EMD approach. Next, the KRLST is used to track the decomposed intrinsic mode functions, and the residual signal is predicted using the LSTM sub-model. Finally, all the predicted intrinsic mode functions and the residual are ensembled to get the future capacity. The experimental and comparative analysis validates the high accuracy (RMSE of 0.00103) of the proposed ensemble approach compared to Gaussian process regression and LSTM fused model. Furthermore, two times lesser error than other fused models makes this approach an efficient tool for battery health prognostics.

## Highlights

Ensemble KRLST-LSTM framework for battery capacity prediction
KRLST trained to address the local regeneration issue of batteries
LSTM utilized to predict the residual
Ensemble KRLST-LSTM increases the prediction accuracy two to three-time

## Graphical abstract

**Figure:**
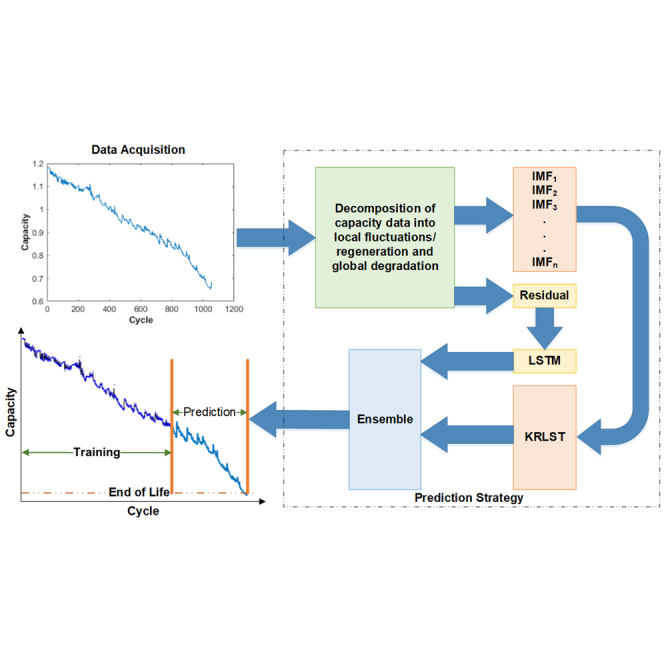

## Introduction

Deterioration in the fossil fuel resources and problems related to climate change provides an excellent stimulus for the developers for focusing on green energy resources, green transportation (i.e., electric vehicles (EVs), hybrid EVs, etc.), and smart grids (Hu et al., 2020; Ahmed et al., 2021). EVs and renewable energy resources will play an essential role in bending the greenhouse gas emission curve for climate mitigation (Creutzig et al., 2016). For the mission of zero-carbon cities, energy storage devices have a significant role (Samadzadegan et al., 2021). Owing to the high energy and power density, low self-discharge rate, and high life cycle (Hu et al., 2020; Mannan et al., 2021), lithium-ion batteries (LIBs) have emerged as the leading power source to actuate all the variants of EVs (Ali et al., 2019a; Khan et al., 2021). The remaining useful life (RUL) and capacity degradation prediction in all LIB applications are demanding tasks (Ali et al., 2019c). The maximum electrical energy, which a battery can store, is known as battery capacity. Because of frequent charging and discharging cycles of LIBs, the battery capacity degraded until its end of life (Umair Ali et al., 2018). Subsequently, the power and charge handling capacity of LIBs drop exponentially. Usually, the battery must be replaced before 20% degradation of its rated capacity to avoid operational failures (Yu, 2018). Therefore, developing a smart battery health prognostic system (SBHPS) for a smooth and reliable battery operation is essential.

Capacity monitoring and RUL prediction are one of the main functions of SBHPS. As the LIB is a highly non-linear system, it is not easy to estimate these parameters. Till to date, several methodologies for RUL and capacity prediction have been reported (Hu et al., 2020; Ali et al., 2019c). Based on the research, these approaches can easily be categorized as model-based, data-driven, and hybrid methods.

In the model-based method, algebraic and differential equations define the physics and the failure mode of LIBs. In a study (Bloom et al., 2001), the authors presented the empirical model to determine the capacity of the LIB. In another study (Cugnet et al., 2009), a fractional-order model is established to correlate the battery crank ability to its resistance. The state of health and state of charge was predicted using the extended Kalman filter and Lagrange multiplier method (Tang et al., 2014; Ali et al., 2019b). Some hybrid methods have also been implemented to estimate the capacity and RUL of the LIB (Guha and Patra, 2017). However, these methods show some excellent results but still have several issues. It is not easy to adjust the LIB parameters for the whole cyclic process (Liu et al., 2020). However, this issue can be eliminated by updating the parameters using adaptive filters, but at a high computational cost. Furthermore, the noise and uncertain environment also affects the estimation accuracy.

The data-driven approaches do not need any prior information on the degradation process. To build the model, it only extracts the useful feature from measurable battery states (i.e., voltage, current, temperature). The machine learning models (i.e., support vector machine (Goebel et al., 2008), Bayesian prediction model (Ng et al., 2014), and neural networks (Wu et al., 2016)) are used to establish a connection between sensor data and battery health. However, most studies ignored the LIB's self-regeneration phenomena, which is a slight fluctuation in the capacity degradation curve because of electrochemical cell relaxation (Zhou and Huang, 2016). Richardson et al. (Richardson et al., 2017) presented the Gaussian process regression (GPR) model to track the local regeneration phenomena. In a study (Yu, 2018), the author utilized a multiscale methodology to estimate and predict the state of health and RUL, respectively. First, the empirical mode decomposition (EMD) method was used to split the LIB capacity. Then, the logic regression and GPR were utilized to form the model. The results showed a significant error in early RUL prediction. Recently (Liu et al., 2020), the long short-term memory (LSTM) and GPR-based fused approach are proposed to track the battery's global degradation and local regeneration, respectively. The result of global regeneration shows high accuracy using LSTM. However, it is difficult to capture the local regeneration phenomena due to high nonlinearity. Recently, a nonlinear kernel-based recursive least square tracker (KRLST) was utilized to track the highly nonlinear signal of electromyogram (Bakshi et al., 2018) and electrocardiogram (Tayel et al., 2018). Therefore, it is meaningful to check the accuracy of KRLST for battery state estimation and prediction.

Driven by the benefits of LSTM and KRLST, this paper proposed a new approach for estimating and predicting the capacity and RUL of the LIB. To be specific, the major contributions of the proposed methodology are the following:

1. The advantage of EMD is that it is used to decompose the nonlinear LIB degradation data into intrinsic mode functions (IMFs) (local regeneration) and residual (global degradation).
2. The KRLST based model is designed for the q-steps ahead prediction of the IMF signals.
3. The residual signal is captured using the LSTM trained sub-model.
4. In the end, both the predictions are ensembled to get the predicted capacity and RUL.
5. Various data-driven models are analyzed and compared to check the performance of the proposed model. The results prove the viability of the proposed ensemble model for SBHPS design.

## Results

**Figure 1:**
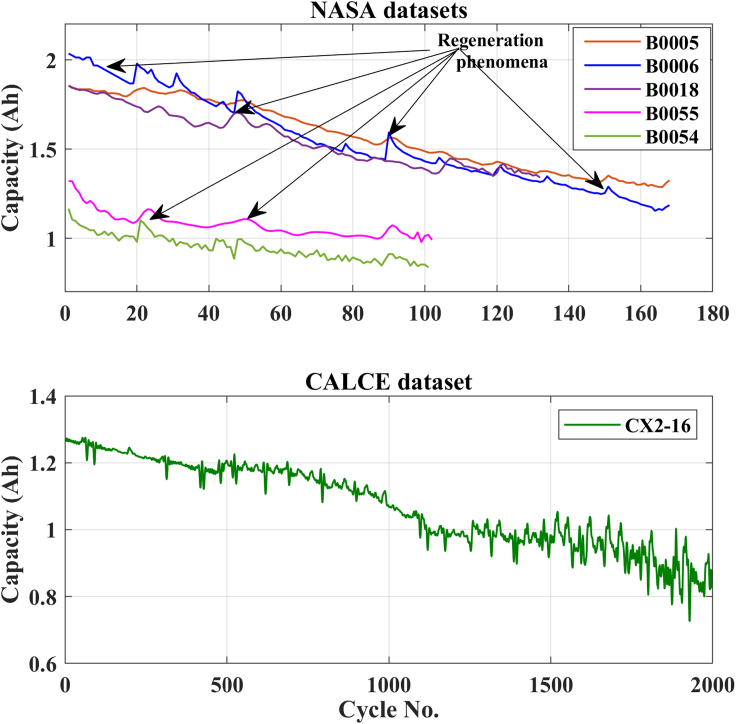
LIB capacity degradation curve of different datasets Saha and Goebel, 2007; Pecht, 2017

The available online datasets (i.e., NASA and CALCE) are utilized to validate the proposed scheme (Saha and Goebel, 2007; Pecht, 2017). The details of the dataset are provided in Table 1. MATLAB 2021 is utilized to perform all the processing. The specification of the personal computer is Intel(R) Core (TM) i7-10700 CPU @ 2.90GHz processor with 32 GB RAM, 1 TB SSD, and a 64-bit Windows 10 Pro operating system (OS). The capacity degradation behavior of all the LIBs used in this work is illustrated in Figure 1.

**Table 1:** Details of available online datasets (Saha and Goebel, 2007; Pecht, 2017)

Battery no.	Type of the battery	Rated capacity (Ah)	Upper cutoff voltage (V)	Lower cutoff voltage (V)	Total no. of cycle	End of life
B0005	Li-ion 18,650	1.86	4.2	2.7	168	127
B0006	Li-ion 18,650	2.04	4.2	2.5	168	127
B0018	Li-ion 18,650	1.85	4.2	2.5	127	97
B0055	Li-ion 18,650	1.32	4.2	2.5	102	70
CX2-16	CS2	1.27	4.2	2.7	2000	1760

For all the NASA batteries (B0005, B0006, B0018, B0054, and B0055), the cyclic aging tests were performed with a programmable electric load, controllable temperature chamber, and power supply (Saha and Goebel, 2007). The operating temperature of 24°C was set for the batteries B0005, B0006, and B0018, all the batteries were cycled at a constant discharge current of 2 A. The operating temperature of the battery (B0055) was set at 4°C, the fixed load current of 2 A was used to cycle the batteries. For the CALCE battery dataset (CX2-16), the Arbin BT2000 system with a temperature-controlled chamber was utilized to conduct all the cyclic tests. The CX2-16 battery was discharged with a constant current of 1.1 A, see (Yu, 2018; Liu et al., 2020) for more details about the experimental setup.

**Figure 2:**
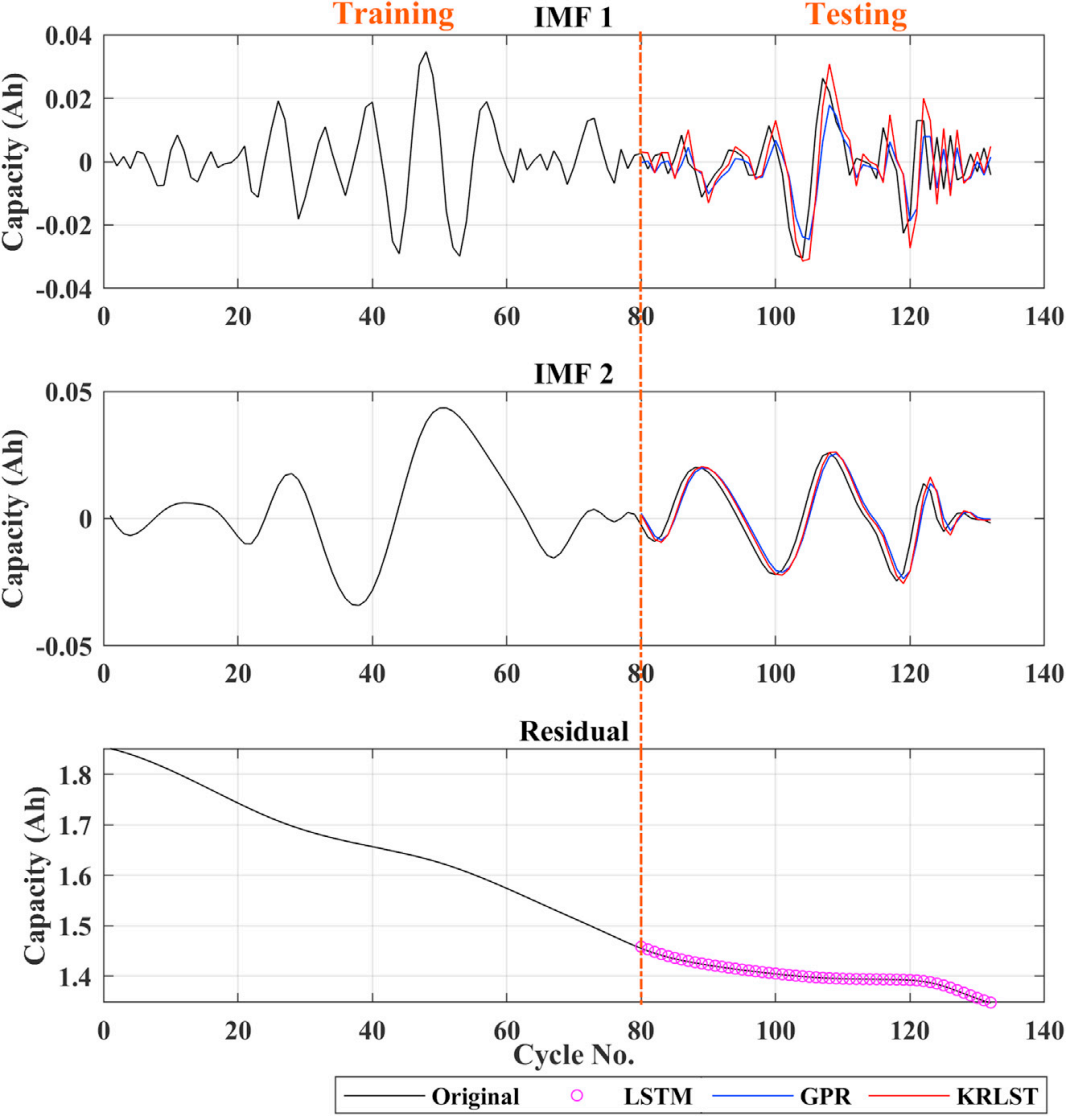
Capacity prediction results of battery B0018

The capacity prediction result of the LIB (B0018) using GPR + LSTM and the proposed technique (KRLST + LSTM) are presented in Figure 2. The model is trained using 80 data from the battery degradation curve of B0018, the remaining data is utilized for the online prediction of the model, as previously used in Ref (Liu et al., 2020).

**Figure 3:**
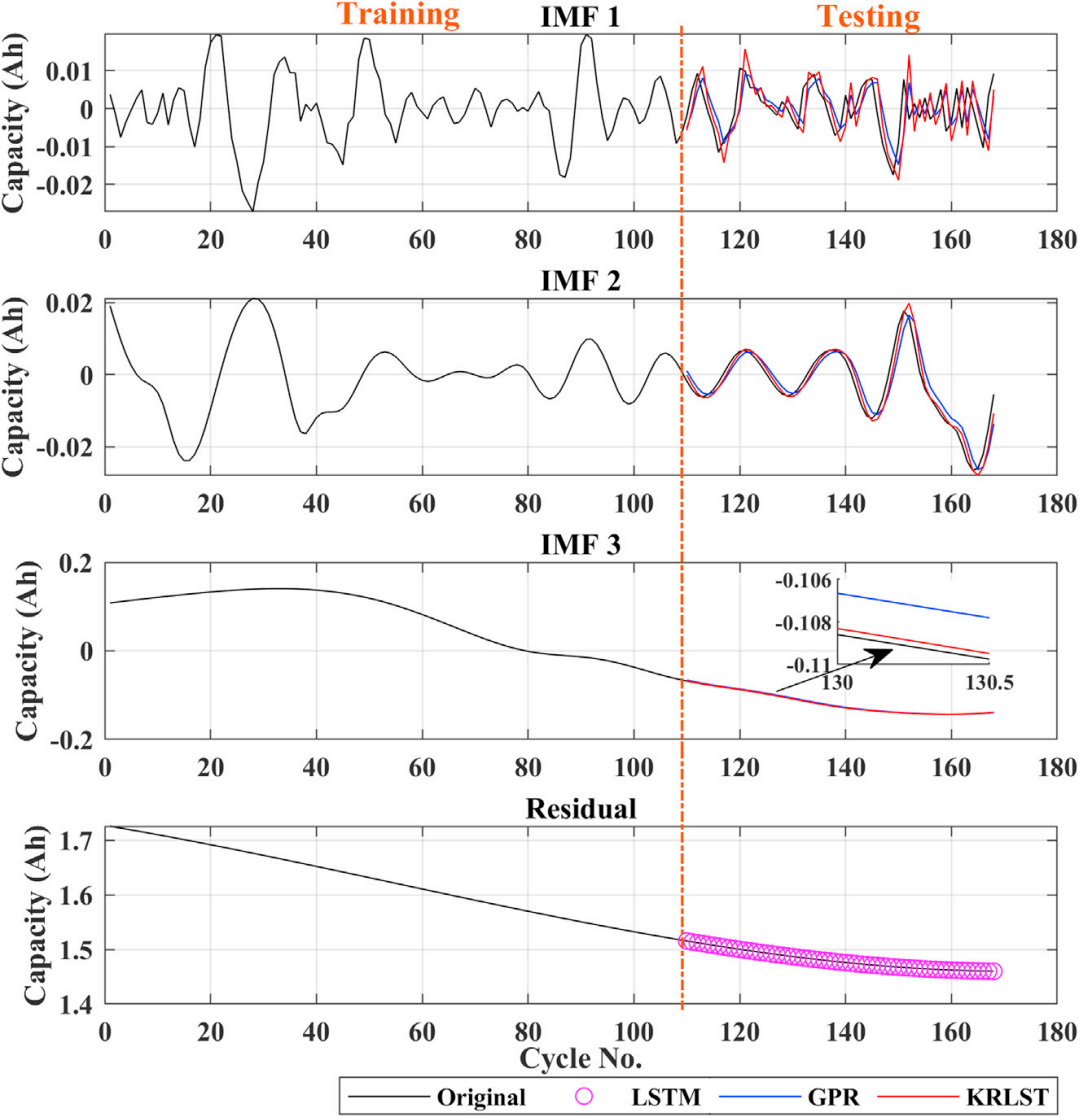
Capacity prediction results of battery B0005

**Figure 4:**
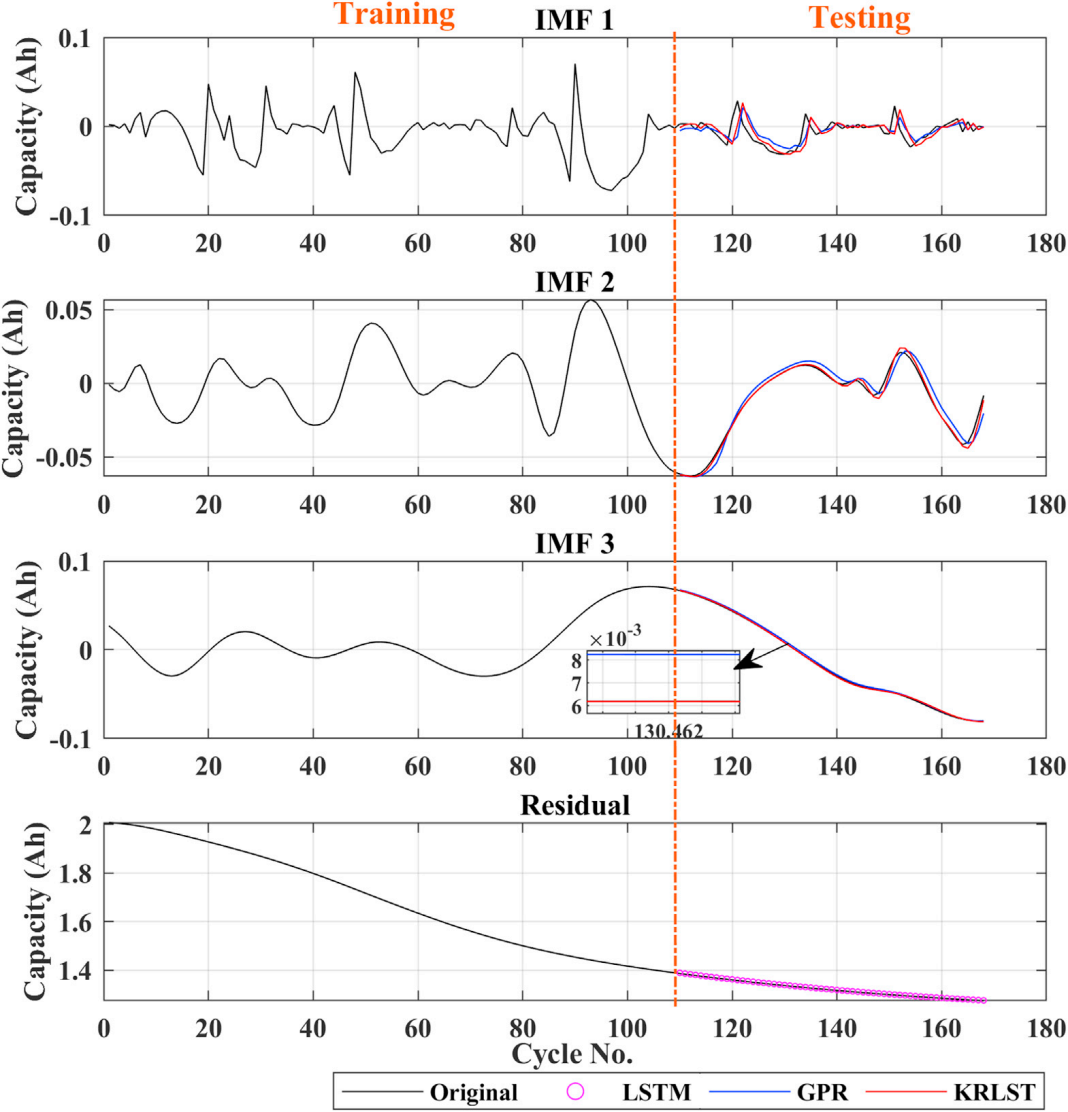
Capacity prediction results of battery B0006

**Figure 5:**
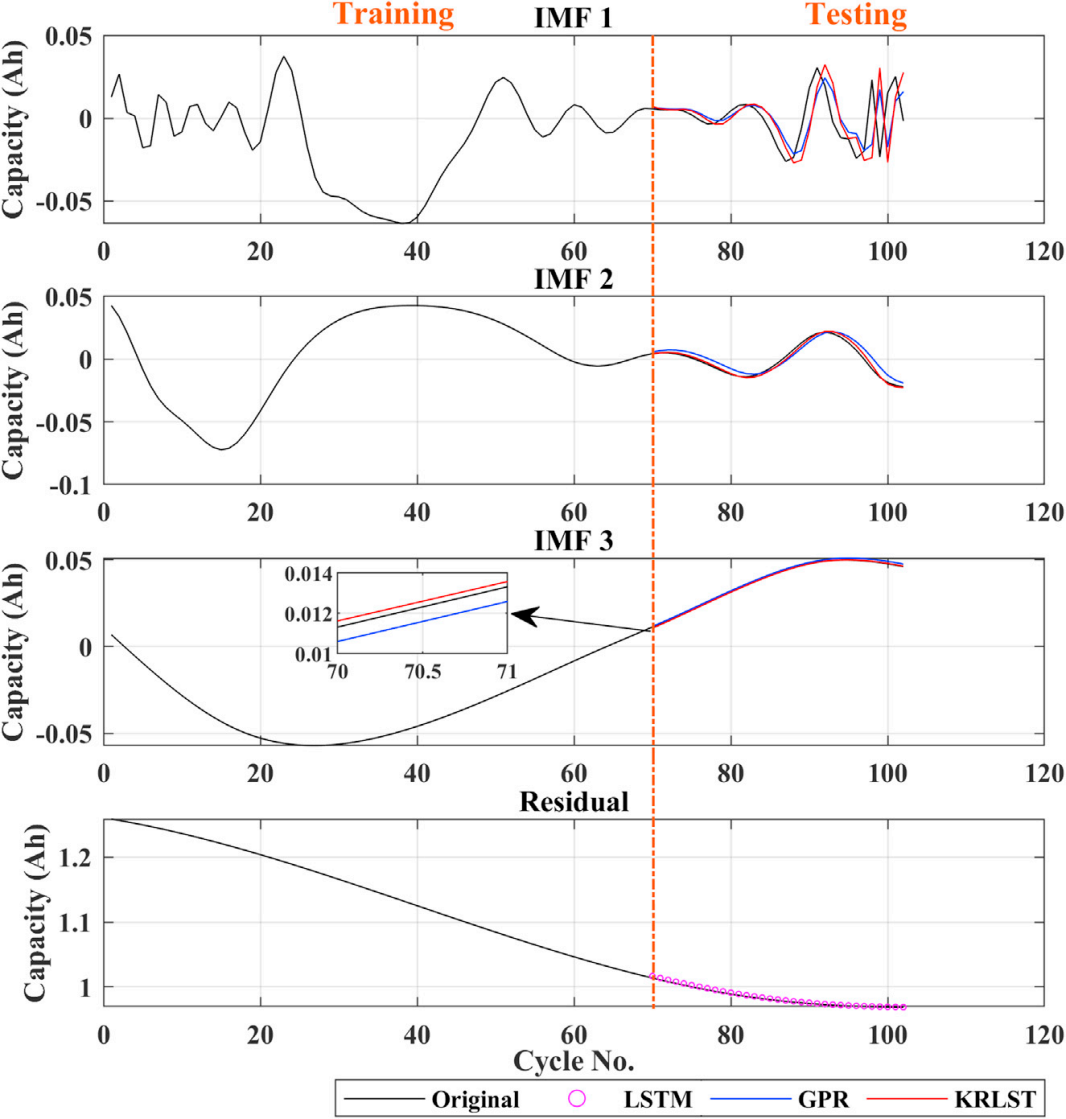
Capacity prediction results of battery B0055

**Figure 6:**
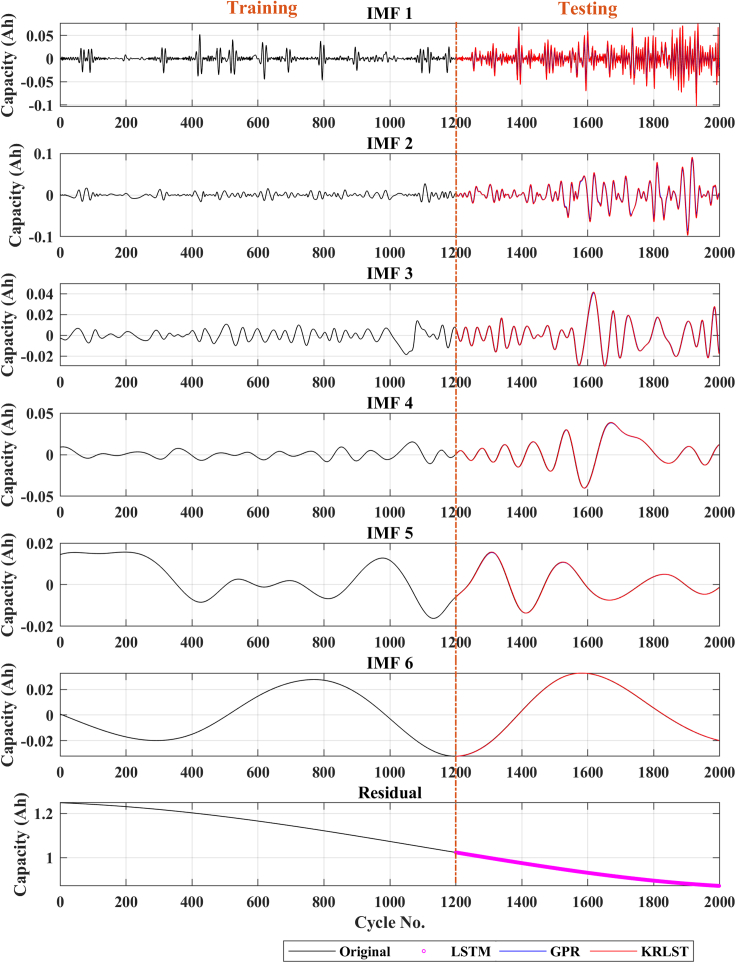
Capacity prediction results of battery CX2-16

To further validate the superiority of the proposed technique, other capacities discharging profiles were also predicted using the proposed and GPR + LSTM approach. The tracking ability and their corresponding RMSE have been shown in Figure 3, Figure 4, Figure 5, Figure 6. 110 capacity data is utilized to train the B0005 and B0006, whereas 70 capacity data is used to train B0055. Remaining battery capacity data is used for online prediction.

The quantitative analysis (i.e., RMSE) of the capacity prediction for the proposed and GPR + LSTM is presented in Table 2. The performance of the proposed methodology is also checked for q-steps ahead prediction. The results of 6, 12, and 18 steps ahead prediction are shown in Table 3.

**Table 2:** Comparison of RMSE of the proposed and GPR-LSTM

Parameter	B0005		B0006		B0018		B0055		CX2-16	
	Proposed	GPR + LSTM	Proposed	GPR + LSTM	Proposed	GPR + LSTM	Proposed	GPR + LSTM	Proposed	GPR + LSTM
IMF1	1.2 × 10^−4^	2.24 × 10^−4^	1.11 × 10^−4^	9.28 × 10^−4^	4.16 × 10^−5^	1.5 × 10^−3^	2.81 × 10^−4^	7.61 × 10^−4^	1.72 × 10^−4^	6.49 × 10^−5^
IMF2	7.52 × 10^−5^	3.46 × 10^−4^	3.53 × 10^−4^	1.28 × 10^−3^	9.96 × 10^−5^	1.6 × 10^−4^	2.38 × 10^−4^	2.44 × 10^−3^	6.16 × 10^−5^	2.37 × 10^−4^
IMF3	1.22 × 10^−4^	1 × 10^−3^	8 × 10^−5^	1.28 × 10^−3^	–	–	2.97 × 10^−4^	6.27 × 10^−4^	1.46 × 10^−4^	1.81 × 10^−4^
IMF4	–	–	–	–	–	–	–	–	9.74 × 10^−5^	8.95 × 10^−4^
IMF5	–	–	–	–	–	–	–	–	5.63 × 10^−5^	2.87 × 10^−4^
IMF6	–	–	–	–	–	–	–	–	1.54 × 10^−4^	3.51 × 10^−4^
Residual	0.00018	0.00018	0.00188	0.00188	0.00088	0.00088	0.00147	0.00147	0.00173	0.00173
Overall	0.00051	0.00176	0.00242	0.00536	0.00102	0.00254	0.00229	0.00530	0.00242	0.00375

**Table 3:** RMSE of proposed technique at q-steps ahead prediction

Battery no.	6-Steps	12-Steps	18-Steps
B0005	0.00113	0.00129	0.00153
B0006	0.00284	0.00296	0.00310
B0018	0.00154	0.00151	0.00182
B0055	0.00295	0.00332	0.00355
CX2_16	0.00273	0.00252	0.00323

**Figure 7:**
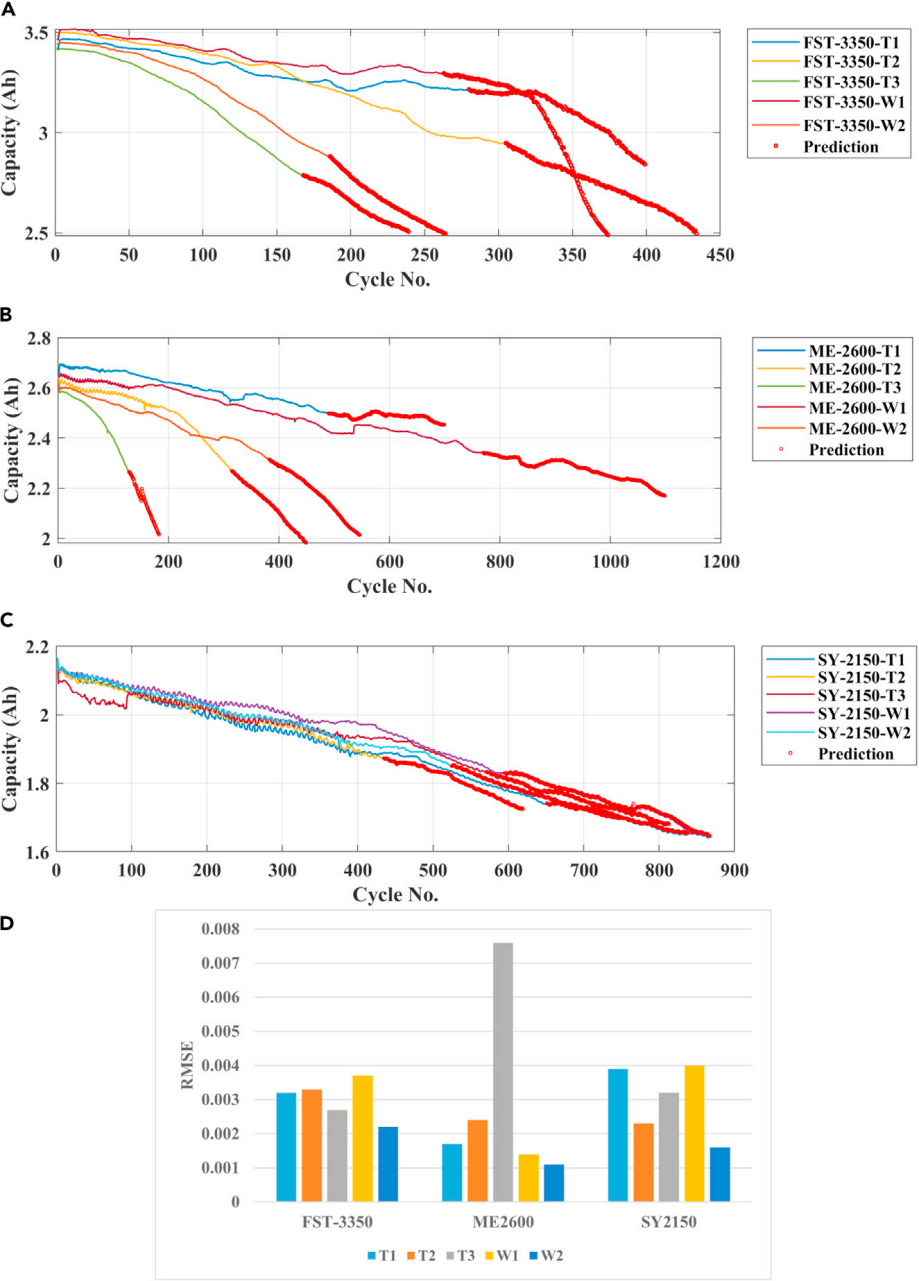
Capacity prediction of batteries using proposed approach (A–D) (A) FST-3350, (B) ME-2600, (C) SY-2150, (D) RMSE.

For further validation of the proposed methodology, a recently published dataset is also utilized to check the effectiveness (Tang et al., 2021). Three batteries of various capacities i.e., 3.35 Ah, 2.600 Ah, and 2.15 Ah namely FST-3350, ME-2600, and SY-2150, respectively are used for the capacity prediction. All the batteries were cycled using constant current and voltage for charging and constant current for discharging. The FST-3350 is discharged at various discharge rates such as 0.3C for W1 and T1, 0.4C for T2, and 0.5C for W2 and T3. Similarly, the discharge rates for ME-2600 are 0.48C, 0.67C, 0.29C, 0.67C, and 0.77C for W1, W2, T1, T2, and T3, respectively. The results of all three batteries are illustrated in Figure 7. Finally, the results of RUL prediction using the proposed ensemble model are reported in Table 4 for NASA and CALCE dataset. The uncertainty (i.e., represented by standard deviation in Table 4) in the RUL prediction is calculated by running the proposed approach ten times for the same cycle.

**Table 4:** RUL prediction results using KRLST and LSTM for NASA and CALCE dataset

Battery no.	Predicted RUL	Actual RUL	Error	Standard deviation
B0005	76	77	1	±3
B0006	47	49	2	±3
B0018	47	47	0	±2
B0055	23	20	−3	±4
CX2-16	247	250	3	±5

## Discussion

In this study, an SBHPS is proposed to avoid the unwanted failure of the LIBs. The SBHPS must be capable enough to predict the accurate RUL of the LIB well before its end of life.

The degradation curves of different online available LIB datasets are illustrated in Figure 1. The capacity degradation showed a non-monotonic behavior over the whole cyclic process. Various local regeneration phenomena and fluctuations can be noted over the cycle number. According to (Richardson et al., 2017), local regeneration and fluctuations commonly occur in all real-time applications. In the past, several tracking algorithms were used to predict the RUL (Liu et al., 2020; Yu, 2018). Zhou et al. (Zhou and Huang, 2016) used the EM technique to decompose the capacity data in several IMFs and a monotonic residual value. They built a GPR sub-model to predict the IMF signals, and the residual signal was predicted using LSTM. In contrast, in this paper, KRLST and LSTM models are used to track the IMF and residual signals, respectively. The 1-step ahead capacity prediction results are illustrated in Figure 2 (for the B0018 battery). The EMD decomposes the degradation signal into 2 IMFs and a single residual signal. The data of 80 cycles out of 127 were used to train the models. In Figure 2, the KRLST tracked the IMF signal with better accuracy than the GPR. The KRLST tracks the IMF battery capacity signals that exhibit nonlinear relationships by forgetting past information and tracking variations in the target latent function. The prediction RMSE of only 0.000041 is noted for IMF 1, whereas the RMSE of the GPR model is 0.0015 (see Table 2). The proposed approach has almost 2-time lesser capacity prediction RMSE than GRP + LSTM. To further validate the dominance of the proposed technique, more datasets are used. Figure 3, Figure 4, Figure 5, Figure 6 and Table 2 show the results of B0005, B0006, B0055, and CX2-16 LIBs, respectively. For B0005 and B0006, the first 110 cycles are utilized to train the sub-models, and the other 58 cycles are used for validation. The tracking efficiency of KRLST is not just high for IMF 1 but also has lesser RMSE for IMF 2 and 3 (see Figure 3 and Table 2). The overall RMSE of 0.00243 is noted to predict the accuracy of B0006 (see Table 2). In Figure 5, the perturbed dataset (discussed in (Yu, 2018)) of B0055 also showed the same accuracy. The LIB with a high life cycle is also tested (see Figure 6). The overall RMSE of 0.00243 is found for the whole prediction process using the proposed approach, which is almost 30% lesser than the proposed one GPR + LSTM (see Table 2). For multi-step ahead prediction, the performance of the proposed method is checked against various prediction horizons of 6, 12, and 18 steps (see Table 3). It is noted that the performance decreases as compared to the 1-step because of the lack of prior information for the future local fluctuations with the increase in the step size. The maximum RMSE of 0.00355 is recorded against the battery B0055 for 18-steps ahead prediction. It is because of high local fluctuation in the B0055 capacity degradation profile, as seen in Figure 1. However, all these RMSE are under 0.004, which indicates the satisfactory performance of the proposed model for 18-steps ahead prediction.

Furthermore, a recently published dataset is also used to prove the performance of the proposed model. The dataset contains three different batteries (FST-3350, ME-2600, and SY-2150) cyclic data. The 70% of capacity data is utilized to train the models, and the remaining dataset is used to predict the model. In Figure 7A, the best prediction performance of the proposed model is noted against the FST-3350-W2 with the RMSE of only 0.0022, whereas the maximum RMSE of 0.0037 is noted against FST-3350-W1 (see Figure 7D). The minimum RMSE of 0.0011 is noted for ME-2600-W2 for this dataset (see Figures 7B and 7D). A similar trend is found for SY-2150 (see Figures 7C and 7D), where the maximum noted error is 0.004 for SY-2150-W1.

In real-world applications, early prediction of accurate RUL is one of the critical roles of SBHPS. The recursive RUL prediction performance of the proposed technique (KRLST and LSTM) is tested, and the findings are presented in Table 4. For B0005, the RUL is predicted at the 50^th^ cycle. If the starting point of RUL prediction is earlier, then prediction performance decreases, as reported by (Mao et al., 2020). The value of the predicted RUL was 76, which is just one cycle earlier than the original value. When the RUL prediction was implemented at the 65^th^ cycle, the error of just three cycles is found for B0006. In B0018, the 100% RUL prediction accuracy was observed for B0018 at the 50^th^ cycle. For B0055, it is observed that the predicted RUL was only three cycles later than the actual RUL. Similar accuracy of the proposed technique can be seen for the CX2-16 LIB. In the case of uncertainty in RUL prediction (see Table 4), a significant improvement in the uncertainty is noted as compared to (Liu et al., 2020). The uncertainty improvement is because the RUL is predicted using the residual (global degradation) of the LIB in this work.

After extensive testing, it can be concluded that the proposed approach has high adaptability and shows high performance for all types of dataset. The reason is that the KRLST has the best tracking capability for time-varying regression. Furthermore, it explicitly handles uncertainty about the data based on the probabilistic GP framework; therefore, it handles the variation in IMFs well. Similarly, LSTM has also shown promising results in the prediction of residual value. Thus, the fusion of the characteristics of these two methods yielded the least RMSE compared to other methods. Moreover, the training time of the presented ensembled model is less than one minute, which means that the ensembled model can be beneficial to design a battery management system for a real-time application. As the Li-ion 18,650 and CS2 batteries are used in cell phones, notebook, and pads; therefore, the proposed model can be used to enhance the prediction of their capacity and RUL. The proposed methodology can be implemented to predict the RUL of the battery in EVs, which leads us toward our goal of zero-carbon cities.

### Conclusions

This study presents a data-driven technique to enable an accurate health prognosis for LIBs. A whole health prediction model of LIB is developed, where various algorithms (EMD, KRLST, and LSTM) are ensembled to perform multiple tasks. The EMD approach decomposes the battery capacity data in IMFs and monotonic residual signals. The KRLST trained model is used to track the local regeneration/fluctuation (IMF), whereas global capacity degradation is predicted using the LSTM trained model. For validation, the proposed and GPR + LSTM approaches are implemented on three different types of available online datasets. The comparative analysis shows that the KRLST has better IMFs tracking ability as compared to the GPR model. The maximum RMSE of 0.00243 for 1-step ahead future capacities are noted against the NASA and CALCE datasets. The proposed model has shown high accuracy for 18-steps ahead of prediction with a maximum RMSE of only 0.00355. The ensembled model also shows high RUL prediction accuracy.

### Limitations of the study

The developed battery health prediction can be utilized to design a battery management system. However, the proposed prediction model is validated through constant conditions (charging current, discharging current, and temperature). Whereas, in most battery-powered applications, the operation conditions vary greatly during cycles, resulting in the battery's multiple degradation modes. So, further research is needed for RUL prediction in a dynamic environment. Furthermore, this work only focuses on the RUL prediction of a single battery cell. In contrast, in many applications such as EV, there are many cells connected in series, parallel, or series and parallel to form a battery pack. The battery pack RUL prediction must be explored because of the uneven aging of the battery cells because of the temperature gradient. In addition, the effects of incorporating optimization algorithms and the variant of EMD in the proposed model can be checked in the future.

## STAR★Methods

### Key resources table

**Table:** 

REAGENT or RESOURCE	SOURCE	IDENTIFIER
**Deposited data**		

"Battery Data Set", NASA Ames Prognostics Data Repository	B. Saha and K. Goebel (2007)	https://ti.arc.nasa.gov/tech/dash/groups/pcoe/prognostic-data-repository/
Battery Data Set. CALCE. CALCE Battery Research Group, Maryland, MD.	Pecht, M. (2017)	https://web.calce.umd.edu/batteries/data.htm
Recovering large-scale battery aging dataset with machine learning	Tang et al. (2021)	https://doi.org/10.1016/j.patter.2021.100302

**Software and algorithms**		

Kernel Adaptive Toolbox MATLAB™	Van Vaerenbergh, (2017)	https://ch.mathworks.com/matlabcentral/fileexchange/46747-kernel-adaptive-filtering-toolbox

### Resource availability

#### Lead contact

Further requests for information should be directed and will be handled by the corresponding author and lead contact, Prof. Dr. Amad Zafar, email: amad.zafar@ee.uol.edu.pk.

#### Materials availability

This study did not generate new materials.

### Method details

The proposed approach consists of EMD, KRLST, and LSTM explained in detail in this section.

#### Empirical mode decomposition

The regeneration and global degradations are the high frequency (IMFs) and low-frequency signal (residual) for LIB capacity. The EMD is a powerful and effective signal processing tool that analyzes the dynamical signal's local characteristics. It decomposes the nonlinear signal into IMFs and residual signals. The conditions for IMFs signal are following; i) the upper and lower envelopes' mean must be equal to zero, ii) the no. of zero crossings, and the no. of extremes must be equal to one or zero.

After computing the input signal's (*x*_*t*_) extreme values, the lower (*Ε*_*t*,*l*_) and upper (*Ε*_*t*,*u*_) envelopes are developed using a spline line. Calculate the mean using the following:

(Equation 1) $$mean_{t}=E_{t,u}+E_{t,l}/2$$

The difference (*diff*) between input and means can be calculated as;

(Equation 2) $$diff=x_{y}-mean_{t}$$

If it is an IMF signal, remove it to get the residual value by using the following equation.

(Equation 3) $$r_{t,1}=x_{t}-diff$$

This process continues until the residue meets the stopping criteria; the IMFs and monotonous residue have all the local regeneration and global capacity degradation information, respectively (Zhou and Huang, 2016). To get the original capacity, IMFs and residuals can be added (Huang et al., 1998). In this work, the signal processing toolbox of MATLAB is used to decompose the battery capacity data. The operational flow of the EMD approach is illustrated in Figure S1.

#### Kernel-based adaptive methods

Kernel-based techniques offer the efficient handling of nonlinear problems of different fields. It transfers the highly nonlinear input data into a high-dimensional feature space, known as reproducing kernel Hilbert space (RKHS). The inner product in the feature space can be easily represented using Mercer's condition (Pérez-Cruz et al., 2013).

(Equation 4) $$\kappa \left(x,x^{\prime }\right)=\left\langle \varphi \left(x\right),\varphi \left(x^{\prime }\right)\right\rangle$$

where *κ*(.,.), *x*, and $x^{\prime }$ are the Mercer kernel function and two different data points. The solution of the nonlinear relationship *f*(*x*) of the input data in terms of kernel function can be expressed using Representer theorem as (Smola and Schölkopf, 2004):

(Equation 5) $$f\left(x\right)=\sum_{m=1}^{m}\varphi \left(n\right)\kappa \left(x_{n},x\right)$$

The online and recursive learning methods calculate the error between the estimated and actual value at each time step to adjust their parameters. The main concerns to implement these methods are the computational complexity and the data storage capacity. To address the issue related to complexity and storage size, the KRLST methods can be adopted. The KRLST method includes a unique feature of forgetting to decide on recent data points. The inclusion of this feature reduces the storage size and shows better adaptability in a non-stationary/nonlinear environment (Bakshi et al., 2018).

#### Kernel recursive least square tracker (KRLST)

The KRLST algorithm basically works on the Bayesian inference framework (Van Vaerenbergh et al., 2012a). The output observation model can be described for input data using GPR; for more details, see (Van Vaerenbergh and Santamaría, 2014). In this work, the following assumption has been made to adopt the KRLST. Assume $X_{j}\in R^{M}$ and $y_{j}\in R^{M}$ are the M dimension input and output data collected from the battery for time (*j*). $A_{t}={\left(X_{j},y_{j}\right)}_{j=1}^{t}$ is the available sequential dataset. The capacity and RUL prediction of the battery can be modeled using the sum of nonlinear latent functions.

(Equation 6) $$y_{j}=f\left(x_{j}\right)+\varepsilon_{j},$$

(Equation 7) $$f\left(x_{j}\right)∼GPR\left(m\left(x\right),\kappa \left(x,x^{\prime }\right)\right)$$

(Equation 8) $$\varepsilon_{j}∼M\left(0,\sigma_{j}^{2}\right)$$

where *m*(*x*) and *ε*_*j*_ are the mean and additive noise (with 0 mean and variance $\left(\sigma_{j}^{2}\right)$) functions, respectively. The *m*(*x*) function is mostly assumed 0.

(Equation 9) $$p\left(y|A_{t}\right)=\int p\left(y|f_{t}\right)p\left(f_{t}|A_{t}\right)df_{t}$$

By using Bayesian and conditional probability rules:

(Equation 10) $$P\left(f_{t+q}|A_{t+q}\right)=P\left({\text{f}}_{t},f_{t+q}|A_{t},y_{t+q}\right)=\frac{P\left(y_{t+q}|f_{t+q}\right)P\left({\text{f}}_{t},f_{t+q}|A_{t}\right)}{P\left(y_{t+q}|A_{t}\right)}=\frac{P\left(y_{t+q}|f_{t+q}\right)P\left(f_{t+q}|{\text{f}}_{t}\right)}{P\left(y_{t+q}|A_{t}\right)}P\left({\text{f}}_{t}|A_{t}\right)$$

If the posterior at time step *t* is a known Gaussian distribution $P\left(f_{t}|A_{t})∼M(f_{t}|\mu_{t},\sum_{t}\right)$, then the above equation can calculate the posterior for the new data point. By using the following assumption, the above-mentioned density function can be calculated.

(Equation 11) $$Q_{t}=K_{t}^{-1},q_{t+q}=Q_{t}k_{t+q},\text{ and }\gamma_{t+q}^{2}=k_{t+q}-k_{t+q}^{T}Q_{t}k_{t+q}$$

At time step *t*

(Equation 12) $$P\left(f_{t+q}|f_{q})∼M(f_{t+q}|q_{t+q}^{T}f_{t},\gamma_{t+q}^{2}\right)$$

Using Equation (10), the $P\left(y_{t+q}|A_{t}\right)$ can be used to get the predictive distribution for the capacity and RUL.

(Equation 13) $$P\left(y_{t+q}|A_{t})=\int P(y_{t+q}|f_{t+q}\right)\left(F_{t+q}|f_{t})(f_{t}|A_{t}\right)d{\text{f}}_{t}df_{t+q}=M\left(y_{t+q}|{\hat{y}}_{t+q},{\hat{\sigma }}_{yt+q}^{2}\right)$$

The latent function's new predictive variance for new input is estimated as:

(Equation 14) $${\hat{\sigma }}_{ft+q}^{2}=k_{t+q}+k_{t+q}^{T}\left(Q_{t}\sum_{t}Q_{t}-Q_{t}\right)k_{t+q}=\gamma_{t+q}^{2}+q_{t+q}^{T}h_{t+q}$$

where $h_{t+q}=\sum_{t}q_{t+q}$ and likelihood, and

(Equation 15) $$P\left(y_{t+q}|A_{t+q})=M(y_{t+q}|A_{t+q},\sigma_{m}^{2}\right)$$

By using Equations (11), (12), and (13).

(Equation 16) $$\begin{array}{c} P\left(y_{t+q}|A_{t+q})=M(y_{t+q}|\mu_{t+q},\sum_{t+q}\right)\\ \mu_{t+q}=\left[\begin{array}{l} \mu_{t}\\ {\hat{y}}_{t+q} \end{array}\right]+\frac{y_{t+q}-{\hat{y}}_{t+q}}{{\hat{\sigma }}_{yt+q}^{2}}\left[\begin{array}{l} h_{t+q}\\ {\hat{\sigma }}_{ft+q}^{2} \end{array}\right]\\ \sum_{t+q}=\left[\begin{array}{ll} \begin{array}{l} \sum_{t}\\ h_{t+q}^{T} \end{array} & \begin{array}{l} h_{t+q}\\ {\hat{\sigma }}_{ft+q}^{2} \end{array} \end{array}\right]-\frac{1}{{\hat{\sigma }}_{ft+q}^{2}}\left[\begin{array}{l} h_{t+q}\\ {\hat{\sigma }}_{ft+q}^{2} \end{array}\right]{\left[\begin{array}{l} h_{t+q}\\ {\hat{\sigma }}_{ft+q}^{2} \end{array}\right]}^{T} \end{array}$$

For the new input, the inverse of the kernel matrix can be calculated as:

(Equation 17) $$\kappa_{t+q}^{-1}=Q_{t+q}=\left[\begin{array}{ll} \begin{array}{l} Q_{t}\\ 0^{T} \end{array} & \begin{array}{l} 0\\ 0 \end{array} \end{array}\right]+\frac{1}{\gamma_{t+q}^{2}}\left[\begin{array}{l} q_{t+q}\\ -1 \end{array}\right]{\left[\begin{array}{l} q_{t+q}\\ -1 \end{array}\right]}^{T}$$

For initialization of some parameters following formulas can be used.

(Equation 18) $$\begin{array}{l} \mu_{1}=\frac{y_{1}\kappa \left(x_{1},x_{1}\right)}{\sigma_{m}^{2}+\kappa \left(x_{1},x_{1}\right)}\\ \sum_{1}=\kappa \left(x_{1},x_{1}\right)-\frac{\kappa {\left(x_{1},x_{1}\right)}^{2}}{\sigma_{m}^{2}+\kappa \left(x_{1},x_{1}\right)}\\ Q_{1}=\frac{1}{\kappa \left(x_{1},x_{1}\right)} \end{array}$$

#### Back to the prior (B2P) forgetting methodology

The most crucial feature of KRLST is the forgetting strategy because the most recent data points contain relevant information, and older points may mislead. In this paper, B2P forgetting strategy is utilized (Van Vaerenbergh et al., 2012b). The posterior GP for the whole dataset can be written as;

(Equation 19) $$\left(f\left(x\right)|A_{t}\right)∼GPR\left(k_{t}{\left(x\right)}^{T}Q_{t}\mu_{t},\kappa \left(x,x^{\prime }\right)\right)+k_{t}{\left(x\right)}^{T}Q_{t}(\left(\sum_{t}-K_{t}\right)Q_{t}k_{t}\left(x^{\prime }\right))$$

where *k*_*t*_(*x*) represents the vector of covariance between *x* and all the other bases $x^{\prime }\text{s}$ in the dictionary at time instance *t*. This posterior is added to another new GP, which does not have any capacity information and independent of $f\left(x\right)|A_{t}$. The new posterior may be defined as (Van Vaerenbergh et al., 2012b):

(Equation 20) $$\tilde{f}\left(x\right)\left|A_{t}=af\left(x\right)\right|A_{t}+bn\left(x\right)$$

where *a* and *b* are the constant, which are used to balance the trade-off between $f\left(x\right)|A_{t}$ and $\tilde{f}\left(x\right)|A_{t}$. By using B2P, Equation (19) becomes

(Equation 21) $$\tilde{f}\left(x\right)|A_{t}∼GPR\left(ak_{t}{\left(x\right)}^{T}Q_{t}\mu_{t},\left(a^{2}+b^{2}\right)\kappa \left(x,x^{\prime }\right)\right)+k_{t}{\left(x\right)}^{T}Q_{t}(\left(a^{2}\sum_{t}-a^{2}K_{t}\right)Q_{t}k_{t}\left(x^{\prime }\right))$$

By comparing Equations (19) and (21), ${\overset{⌣}{\mu }}_{t}=a\mu_{t},{\sum^{⌣}}_{t}=a^{2}\sum_{t}+\left(1-a^{2}\right)K_{t},a^{2}+b^{2}=1$. It means that the *a* is the +ve number and has a value in the range of 0–1. By putting the *a*^2^=*λ*, the forgetting updated can be viewed as.

(Equation 22) $$\begin{array}{l} \sum_{t}\leftarrow \lambda \sum_{t}+\left(1-\lambda \right)K_{t}\\ \mu_{t}\leftarrow \sqrt{\lambda }\mu_{t} \end{array}$$

The value of 0.99 is used for*λ* in this work. To ensure numerical stability in real-time applications, a jitter (ζ) noise term is added in KRLST. Thus, the kernel function can be updated as $\left[\kappa \left(x,x^{\prime }\right)+\zeta \right].$ It ζ is not part of the algorithm, but it is the parameters whose value depends on the machine's precision (Pérez-Cruz et al., 2013). The flowchart of the working of KRLST is shown in Figure S2.

#### Long short-term memory (LSTM)

(Hochreiter and Schmidhuber, 1997) introduced the particular type of recurrent neural network (RNN), known as LSTM. The special kind of RNN was typically designed to mitigate the long-term dependency problem caused by the gradient vanishing problem. The incorporation of the gates in LSTM regulates the long-term dependency issue. The basic structure of the LSTM unit can be divided into three gates, as shown in Figure S3.

All the states can be determined by input at the current time step (*x*_*t*_), output at the previous time step $\left(h_{t-1}\right)$, and a sigmoid function (σ(.)). The function of the input gate (*i*_*t*_) is to decide about the receiving of new information $\left({\hat{c}}_{t}\right)$, whereas the forgetting gate (*f*_*t*_) and the output gate (*o*_*t*_)are responsible for deciding whether to forget about the previous state (*c*_*t*−1_) in the hidden layers and determine output (*h*_*t*_), respectively. The following mathematical equations can be used to model the system.

(Equation 23) $$i_{t}=\sigma \left(W_{i}.\left(x_{t},h_{t-1}\right)+b_{i}\right)$$

(Equation 24) $$f_{t}=\sigma \left(W_{f}.\left(x_{t},h_{t-1}\right)+b_{f}\right)$$

(Equation 25) $$o_{t}=\sigma \left(W_{o}.\left(x_{t},h_{t-1}\right)+b_{o}\right)$$

(Equation 26) $${\hat{c}}_{t}=\tanh\left(W_{c}.\left(x_{t},h_{t-1}\right)+b_{c}\right)$$

(Equation 27) $$c_{t}=f_{t}⊗c_{t-1}+i_{t}⊗{\hat{c}}_{t}$$

Equation 28 $$h_{t}=O_{t}⊗\tanh\left(c_{t}\right)$$

where $W_{*}$, $b_{*}$, and ⊗ are the weight matrix, bias vector, and element-wise multiplication (Hochreiter and Schmidhuber, 1997; Liu et al., 2020). The 50 epochs are selected to train the LSTM model.

#### Proposed methodology

In this study, the hybridization of EMD, KRLST, and LSTM has been proposed to predict the RUL of LIBs. The block diagram of the proposed approach is illustrated in Figure S4.

The measured noisy signals (voltage and current) of LIBs are filtered through the Savitzky-Golay filter (Richardson et al., 2018). The MATLAB command *sgolayfilt* is used to implement the filter. The EMD decomposes the capacity data in several IMFs and residual. The KRLST is used to track the nonlinearity of IMFs, whereas the residual signal is predicted through LSTM. The outputs of both the sub-models are ensembled to get the predicted capacity and RUL. Finally, the RMSE is used to check the accuracy of the models.

(Equation 29) $$RMSE=\sqrt{\frac{1}{N}\sum_{i=1}^{N}{\left(y_{t,i}-{\hat{y}}_{t,i}\right)}^{2}}$$

## Data Availability

• All datasets utilized in this study are available online. • This paper does not report original codes. • Any additional information for reanalyzing the work is available from the lead contact upon request.
