# Supplementary material for: Kernel recursive least square tracker and long-short term memory ensemble based battery health prognostic model
Source: iScience. 2021 Oct 15;24(11):103286. doi: 10.1016/j.isci.2021.103286 (PMC8571724; doi:10.1016/j.isci.2021.103286)
Supplement: Document S1. Figures S1–S4 [file mmc1.pdf]

iScience, Volume *24*

Supplemental information

Kernel recursive least square tracker and long-short term memory ensemble based battery health prognostic model

Muhammad Umair Ali, Karam Dad Kallu, Haris Masood, Kamran Ali Khan Niazi, Muhammad Junaid Alvi, Usman Ghafoor, and Amad Zafar

Supplemental Information

Kernel Recursive Least Square Tracker and Long-Short Term Memory Ensemble Based Battery Health Prognostic Model

Muhammad Umair Ali, Karam Dad Kallu, Haris Masood, Kamran Ali Khan Niazi, Muhammad Junaid Alvi, Usman Ghafoor, and Amad Zafar

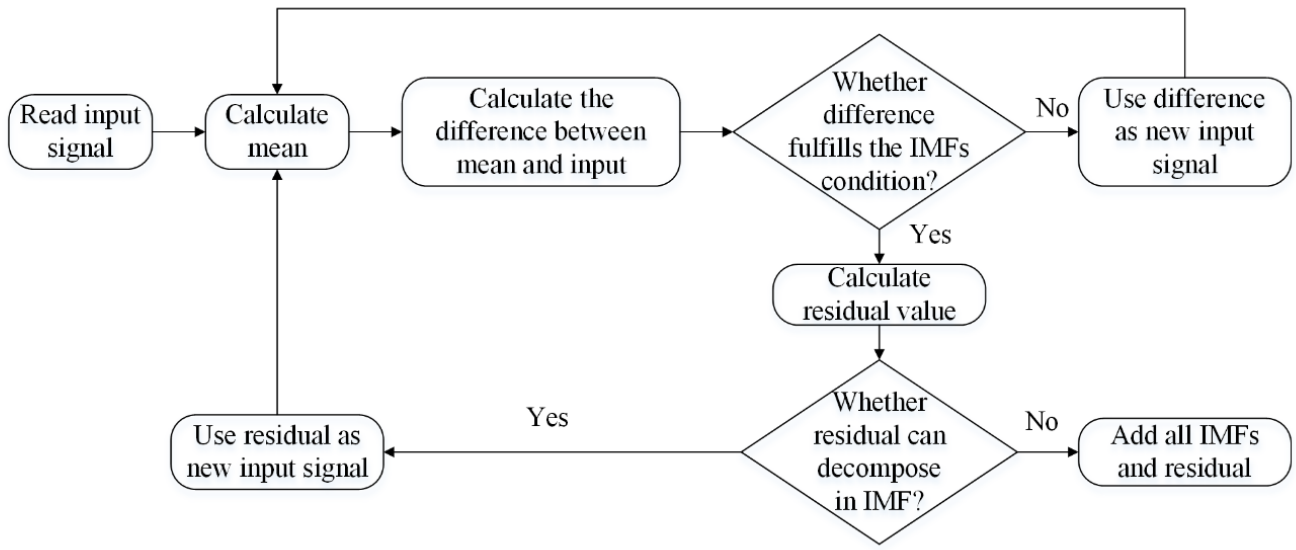

Figure S1. Working flow of EMD technique, Related to STAR Methods.

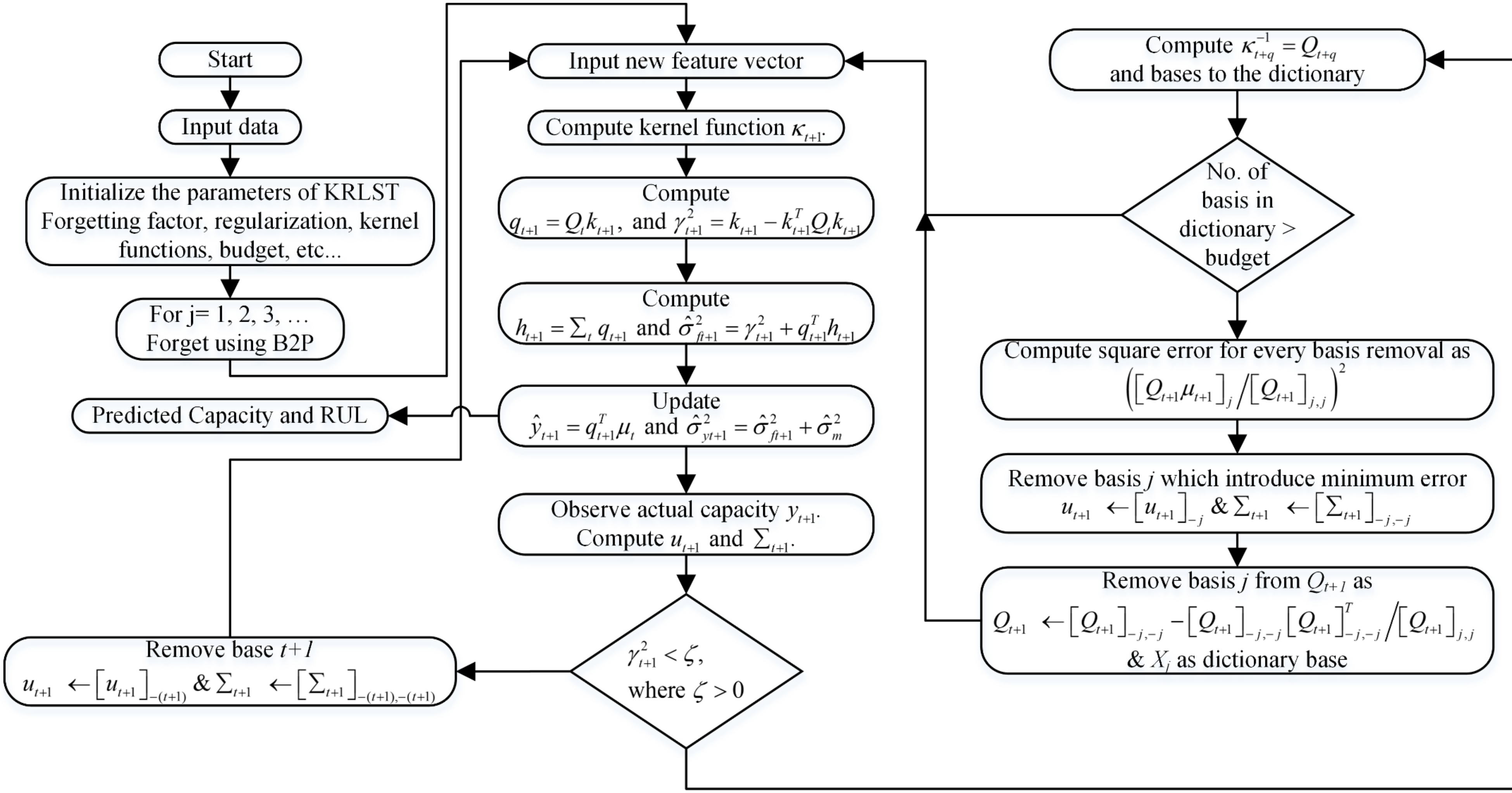

Figure S2: The KRLST framework for prediction of local fluctuation and regeneration of LIBs, Related to STAR Methods.

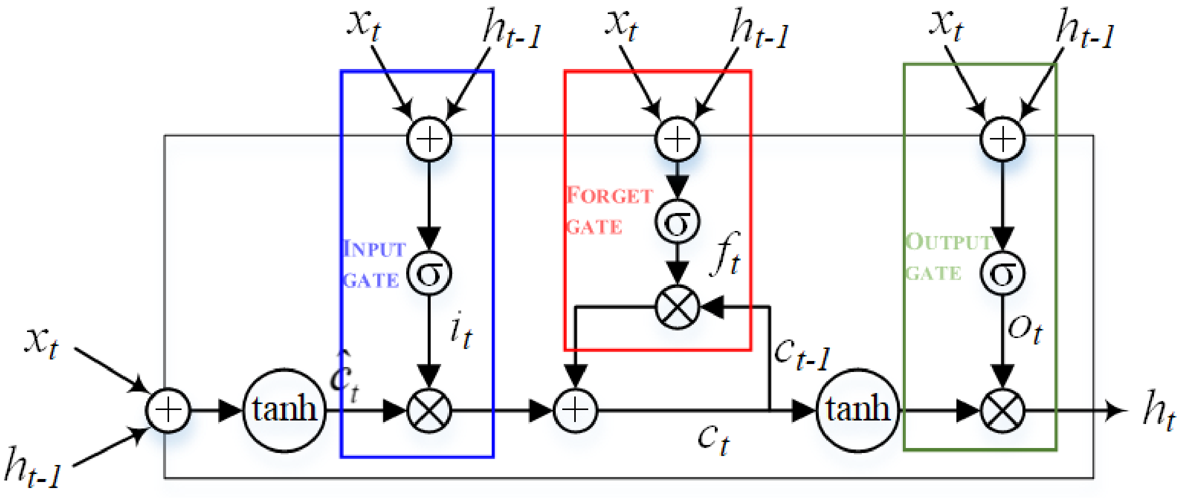

Figure S3: Structure of typical LSTM model, where x_t, h_t, $\sigma(.)$, i_t, f_t, and o_t are the input, output, sigmoid function, input gate, forgetting gate, and output gate, respectively, Related to STAR Methods.

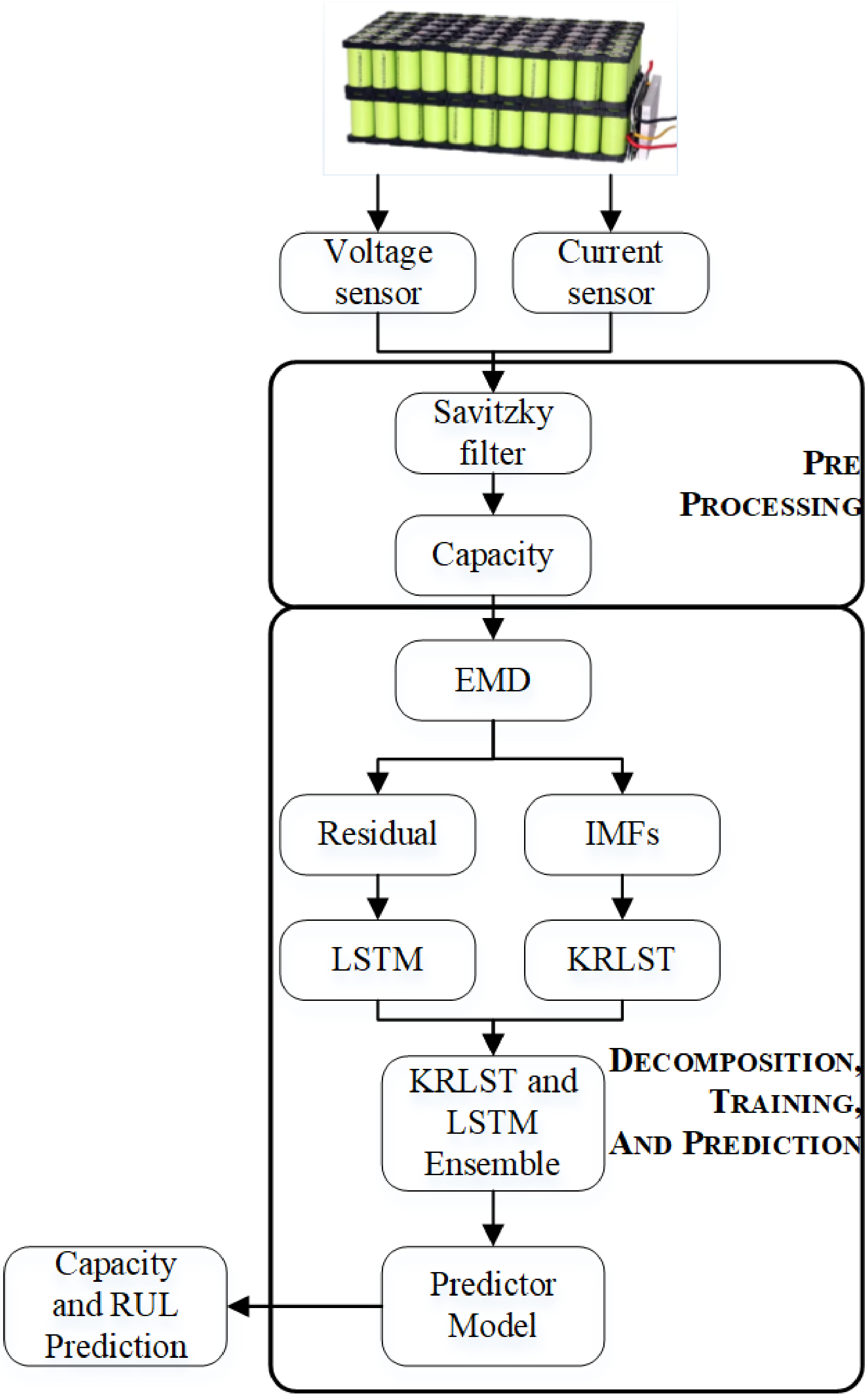

Figure S4. The proposed methodology to predict capacity and RUL of LIBs, Related to STAR Methods.
